# Progress in genome-wide association studies of schizophrenia in Han Chinese populations

**DOI:** 10.1038/s41537-017-0029-1

**Published:** 2017-08-10

**Authors:** Weihua Yue, Xin Yu, Dai Zhang

**Affiliations:** 1 Institute of Mental Health, the Sixth Hospital, Peking University, 100191 Beijing, China; 2 Key Laboratory of Mental Health, Ministry of Health & National Clinical Research Center for Mental Disorders (Peking University), 100191 Beijing, China; 3 Peking-Tsinghua Joint Center for Life Sciences & PKU-IDG/McGovern Institute for Brain Research, Peking University, 100871 Beijing, China

## Abstract

Since 2006, genome-wide association studies of schizophrenia have led to the identification of numerous novel risk loci for this disease. However, there remains a geographical imbalance in genome-wide association studies, which to date have primarily focused on Western populations. During the last 6 years, genome-wide association studies in Han Chinese populations have identified both the sharing of susceptible loci across ethnicities and genes unique to Han Chinese populations. Here, we review recent progress in genome-wide association studies of schizophrenia in Han Chinese populations. Researchers have identified and replicated the sharing of susceptible genes, such as within the major histocompatibility complex, microRNA 137 (*MIR137*), zinc finger protein 804A (*ZNF804A*), vaccinia related kinase 2 (*VRK2*), and arsenite methyltransferase (*AS3MT*), across both European and East Asian populations. Several copy number variations identified in European populations have also been validated in the Han Chinese, including duplications at 16p11.2, 15q11.2-13.1, 7q11.23, and VIPR2 and deletions at 22q11.2, 1q21.1-q21.2, and *NRXN1*. However, these studies have identified some potential confounding factors, such as genetic heterogeneity and the effects of natural selection on tetraspanin 18 (*TSPAN18*) or zinc finger protein 323 (*ZNF323*), which may explain the population differences in genome-wide association studies. In the future, genome-wide association studies in Han Chinese populations should include meta-analyzes or mega-analyses with enlarged sample sizes across populations, deep sequencing, precision medicine treatment, and functional exploration of the risk genes for schizophrenia.

## Introduction

Schizophrenia is a common severe psychiatric disorder that affects ~1% of the world population with high heritability in the range of 64–81% and a complex genetic architecture.^1–3^ Schizophrenia is often characterized as a heterogeneous disorder, and its genetic basis has long been explored using family-based or twin-based studies.^3^ In early studies, linkage and candidate gene association studies implicated numerous putative risk chromosome loci for this disease. However, inconsistent findings resulting from limited biological information on single loci and inadequate sample sizes have sharply hampered the progress of these studies.

**Fig. 1:**
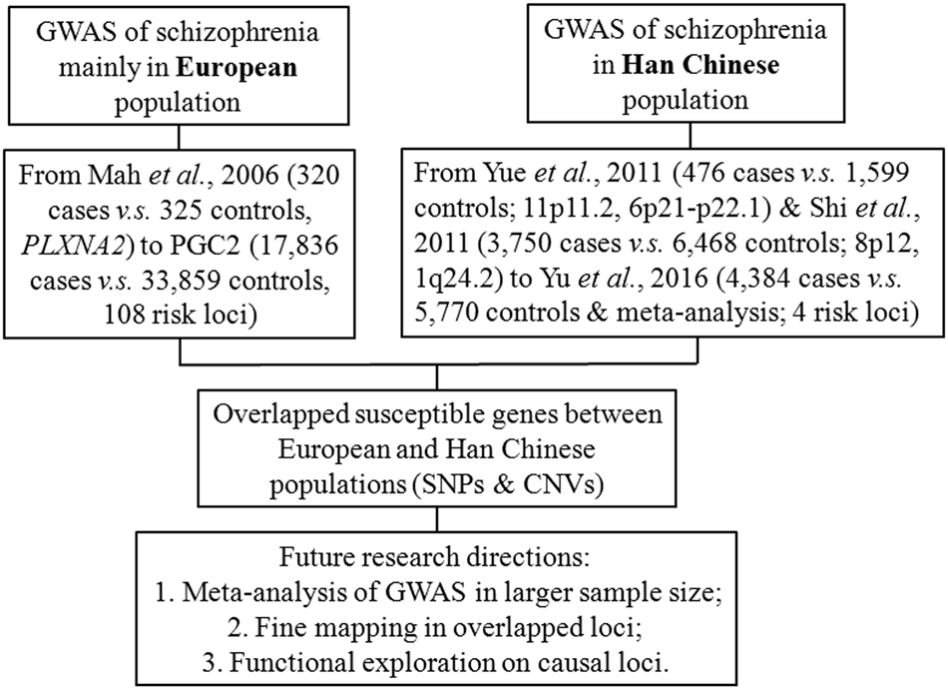
Flow chart unraveling causal genes of schizophrenia from GWAS risk loci across multi-ethnicities

Since 2005, the strategy of genome-wide association studies (GWASs) has rapidly prompted additional studies examining the biological mechanism of other complex diseases. The success of GWASs has typically been evaluated based on the number of susceptible genes or risk loci discovered, representing just one achievement of the GWAS approach. Thus, it should be much more meaningful to replicate or validate the findings of various GWASs and to explain the clinical implications of specifically associated single nucleotide polymorphisms (SNPs). Accumulative associated genes reported by GWASs may shed light on the pathophysiological mechanisms contributing to special disease phenotypes and potential therapeutic targets. However, until recently, most large-scale GWASs focused primarily on Western populations, although other studies have examined more diverse populations. Here, we will review the progress in GWASs of schizophrenia in Han Chinese populations. Figure 1 indicates the flow chart of this review, which unravels causal genes of schizophrenia from GWAS risk loci across multi-ethnicities.

## Progress in GWASs of schizophrenia mainly in European populations

### Common polymorphisms associated with schizophrenia in European Populations

In 2006, Mah et al. reported the first GWAS of schizophrenia, including 320 patients of European descent and 325 matched controls. They revealed that the semaphorin receptor plexin A2 (*PLXNA2*) may be a susceptible locus for this disease in people of European ancestry.^4^ To date, myriad risk loci for schizophrenia have been identified using GWAS approaches.^5–21^ Shifman et al. performed a GWAS for schizophrenia in 660 cases and 2271 controls of the Ashkenazi Jewish population and they identified the risk gene *RELN*. These authors have also validated *RELN* as susceptible loci in four additional populations (2274 cases and 4401 controls), including 415 patients and 458 normal controls of Chinese ancestry.^5^ O’Donovan et al. carried out a GWAS of schizophrenia (479 cases, 2937 controls) in United Kingdom (UK) samples. They then validated suggestive loci in up to 16,726 additional subjects, which included individuals from China and Japan, as well as Ashkenazi Jews and outbred European populations. The number of Chinese individuals was 1034 cases and 1034 normal controls.^6^ In 2009, three GWAS studies from the ISC, SGENE, and MGS consortia individually implicated the major histocompatibility complex (*MHC*) region located on chromosome 6p22.1, transcription factor 4 (*TCF4*) and neurogranin (*NRGN*) as key susceptible genes for schizophrenia at significant levels of *P* < 5 × 10^−8^.^7–9^ These findings also further supported the abnormal immune system and neurodevelopmental hypothesis of this disease. Subsequently, several independent studies have also replicated the MHC findings for schizophrenia across various populations. Moreover, meta-analyzes (combining GWAS results) or mega-analyses (combining GWAS data), which dramatically enlarge the statistical power of a GWAS by pooling subjects, have played important roles in promoting worldwide GWASs of complex diseases into the new so-called “big data era”.^22^ In 2011, Steinberg et al. conducted a meta-analysis in a large replication sample and added vaccinia-related kinase 2 (*VRK2*) as well as replicating the MHC region and *TCF4* associations.^10^

In recent years, the largest and encouraging schizophrenia GWAS came from the Psychiatric Genomics Consortium (PGC). To date, the PGC (http://pgc.unc.edu) has involved more than 900 investigators from 40 countries, with an open-source dataset representing more than 400,000 human participants.^22^ PGC stage 2 (PGC2) has identified five novel loci [1p23.3 (*MIR137*), 2q32.3 (*PCGEM1*), 8p23.2 (*CSMD1*), 8q21.3 (*MMP16*), and 10q24.32-q24.33 (*CNNM2/NT5C2*)] in a large discovery sample of 21,856 individuals of European ancestry and a replication sample of 29,839 independent subjects.^11^ 1 year later, the PGC reported 13 novel schizophrenia loci (*CACNA1C*, *CACNB2*, *ZNF323*, etc.) from a much larger multi-stage GWAS (5001 cases and 6243 controls from Sweden followed by meta-analysis with a previous GWAS of 8832 cases and 12,067 controls and finally by replication of independent samples from 7413 cases, 19,762 controls and 581 parent-offspring trios).^12^ Recently, the PGC2 successfully identified 128 linkage disequilibrium (LD)-independent SNPs in 108 distinct loci using GWASs of 17,836 cases and 33,859 controls of European ancestry.^13^ The susceptible genes include some potential therapeutic targets (such as *DRD2*; glutamate metabotropic receptor 3, *GRM3*) and genes involved in neurodevelopment, glutamatergic neurotransmission (for example, *GRIN2A*, *GRIA1,* and *SRR*), neuronal calcium signaling (for example, *CACNA1C*, *CACNB2,* and *CAMKK2*) and synaptic function and plasticity (for example, *KCTD13*, *CNTN4*, and *MEF2C*).^13^ These findings have provided new insights into the pathogenesis of schizophrenia. Table 1 showed the summary of progress in GWASs of schizophrenia mainly in European populations (Shifman et al. included 415 patients and 458 normal controls of Han Chinese; O’Donovan et al. included 1034 cases and 1034 normal controls).^5, 6^ The plan for PGC3 will be to further enlarge the GWAS sample size to 100,000 cases and at least 20,000 subjects will be sequenced for 200 genes, and analyzes of the Network or Pathway, the PsychENCODE project, etc. will be performed.^22^

**Table 1:** Summary of progress in GWASs of schizophrenia mainly in European populations and two studies in Chinese Han population

Articles	GWAS sample size case/control	Replication sample size case/control	Populations	Susceptible genes
Mah, 2006	320/325	–	European	*PLXNA2*
Shifman, 2008	660 /2271	2274/4401	European	*RELN*
O’Donovan, 2008	479/2937	6666/ 9897	Mutli-ethnicity	*ZNF804A*
Stefansson, 2009	2663/13498	10282/21093	European	*HIST1H2BJ, NOTCH4, NRGN, PGBD1, PRSS16, TCF4*
ISC, 2009	3322/3587	4692/15493	European	*6p22.1, MYO18B, NOTCH4, RPP21*
PGC, 2011	9394/12462	8442/ 21397	European	*MIR137, CACNA1C, ANK3, ITIH3-ITIH4*
Yue, 2011	746/1599	4027/5603	Han Chinese	*TSPAN18, NKAPL, ZKSCAN4, PGBD1*
Shi, 2011	3750/6468	4383/4539	Han Chinese	*LSM1, WHSC1L1 FGFR1*
PGC, 2014	35476/46839	1513/66236	Multi-ethnicity	108 SNPs

### Rare copy number variations (CNVs) of schizophrenia in European populations

Accumulating evidence suggested rare, large CNVs contribute to vulnerability for schizophrenia. To date, most of the evidence of CNVs has been reported from studies in populations with European ancestry. The 22q11.2 deletion was the first CNVs reported as implicated in schizophrenia, and the prevalence of 22q11.2 deletion in patients with SZ is about 0.3%.^23, 24^ However, Rees et al. further showed that duplications of 22q11.2 may protect against schizophrenia.^25^ Moreover, micro-deletions at 1q21.1,^24, 26^ 2p16.3,^27, 28^ 3q29,^27, 29^ 15q11.2,^26, 28^ 15q13.3,^24, 26, 27^ 17p12,^28^ 17q12,^30^ and duplications on 1p36.33,^25^ 15q13.1,^28^ 16p11.2,^25, 29, 31^ 16p13.1,^28, 32^ VIPR2,^27, 33^ and CGNL1^25^ have been identified to be associated with an increased risk of schizophrenia.^34^

### Functional exploration on GWAS-identified loci of schizophrenia in European Populations

Moreover, one of the striking aspects of efforts based on GWASs is to explore the potential functional implications for risk genes of schizophrenia. Last year, two other studies based on large-scale GWASs of schizophrenia were also convincing. Sekar et al. reported that complex variations of the complement component 4 (*C4*) gene may increase the genetic risk of schizophrenia and further explore the potential function contributing to the mechanism of schizophrenia.^19^ Another study identified a human-specific arsenite methyltransferase (*AS3MT*) isoform that may be one of the molecular risk factors in the 10q24.32 schizophrenia-associated locus.^20^

However, the vast majority of susceptibility loci were identified in samples of European ancestry.^4–14, 19, 20^ The associated variants identified in European populations might not be associated with schizophrenia in other ancestry groups because of underlying genetic heterogeneity. Therefore, large-scale studies in non-European populations are necessary not only to investigate whether the previously identified loci can be generalized to non-European populations but also to identify new schizophrenia susceptibility loci. However, the European population accounts for the predominant findings, reflecting relatively larger sample sizes, improved collaborative mechanisms, etc. For example, the PGC3 groups are working on the meta-analyses across East Asian populations, included Shi et al.’s and Yue et al.’s GWAS data.^15, 16^

## Progress in GWASs of schizophrenia in Han Chinese populations

### Common polymorphisms associated with schizophrenia in Han Chinese

Although GWASs of schizophrenia in non-European populations were limited to small sample sizes and have yielded few risk loci during the last 6 years, GWASs of Han Chinese populations have successfully identified both the sharing of susceptible genes of schizophrenia across ethnicities and genes unique to Han Chinese populations. There are many publications using GWASs and further analyzes exploring susceptible genes or loci in Han Chinese populations (Table 2). Most of these studies have focused on the replication or verification of the susceptible genes of schizophrenia in Han Chinese. To identify new common genetic risk factors, two research groups independently conducted GWASs in Han Chinese populations in 2011.^15, 16^ Yue et al. used 479 cases with schizophrenia and 1599 healthy control subjects, followed by 4027 individuals with schizophrenia and 5603 controls as a replication sample, particularly those of Northern Han Chinese descent. These researchers identified a previously unknown variation in a region of chromosome 11p11.2 as a novel susceptible locus for schizophrenia in Han Chinese populations and they also replicated the chromosome 6p22.1 finding from European populations that included the MHC region.^15^ Shi et al. compared the genomes of 3750 schizophrenia patients with those of 6468 controls comprising three subgroups (Northern, Central and, Southern populations). This study revealed two significantly associated regions, 8p12 (Wolf–Hirschhorn syndrome candidate 1-like 1, *WHSC1L1*; LSM1 homolog, mRNA degradation associated, *LSM1*) and 1q24.2 (mitochondrial pyruvate carrier 2, *BRP44*). The association of these two regions with schizophrenia was validated in another sample of 4383 schizophrenia cases and 4539 control subjects.^16^

**Table 2:** GWAS and further analyzes of schizophrenia (SCZ) and related traits in Han Chinese population

Authors and years or publication	Diseases or traits	Sample sizes or datasets^a^	Types of studies	Findings of studies
Li, T. et al. 2010 ^48^	SCZ	2496 SCZ patients and 5184 controls of Chinese Han	Replication of a GWAS signal at *MHC*	*TCF4* not *NRGN* associated with SCZ
Zeng, Z. et al. 2011^38^	Bipolar disorder (BPD), SCZ, and major depressive disorder (MDD)	1139 BPD cases (645 type I); 1122 SCZ cases; 1122 MDD cases and 1138 controls	Meta-analysis and replication of a GWAS signal at *DGKH*	*DGKH* associated with bipolar disorder and SCZ
Ma, X. et al. 2011^65^	Fluid intelligence in SCZ	98 SCZ cases and 60 controls	GWAS of SCZ phenotype	*MSRA* associated with fluid intelligence in SCZ
Yue, W. et al. 2011^15^	SCZ	GWAS: 746 SCZ cases and 1599 controls; Replication:4027 SCZ cases and 5603 controls	GWAS	6p22.1 (*NKAPL, ZKSCAN4, PGBD1*) and 11p11.2 (*TSPAN18*) associated with risk of SCZ
Shi, Y. et al. 2011^16^	SCZ	Discovery: 3750 SCZ cases and 6468 controls; Replication: 4383 cases and 4539controls	GWAS	8p12 (*WHSC1L1*, *LSM1*) and 1q24.2 (*BRP44*) associated with risk of SCZ
Liou, Y.J. et al. 2012^68^	Treatment Refractory SCZ (TRS)	795 TRS cases and 806 controls	GWAS of TRS	*NFKB1* involved in development of TRS
Li, M. et al. 2012^39^	SCZ	10 Chinese and 2 Japanese samples, including 8857 cases and 12,205 controls	Meta-analysis & Replication of a GWAS signal at *ZNF804A*	*ZNF804A* rs359895 not rs1344706 contributes to SCZ in Asian
Yuan, A. et al. 2012^50^	SCZ	516 SCZ cases, 400 controls, and 81 trios with early onset SCZ probands	Replication of a GWAS signal at *ANK3*	*ANK3* associated with SCZ
Ma, L. et al. 2013^37^	SCZ	976 unrelated SCZ cases and 1043 controls	Replication of nine GWAS signals	negative
Zhang, Y. et al. 2013^46^	SCZ	902 cases and 1091 controls	Replication of GWAS signal at 6p21-6p22.1	6p21-6p22.1 associated with SCZ
Yuan, J. et al. 2013^47^	SCZ	1093 SCZ cases and 1022 controls	Replication of a GWAS signal at *TSPAN18*	Replication *TSPAN18* rs835784 ‘A’ as risk allele of SCZ
Li, M. et al. 2013^40^	SCZ	8982 cases and 12,342 controls	Meta-analysis & replication of a GWAS signal at ZNF804A	Negative
Li, M. et al. 2013^41^	SCZ	12,477 cases and 14,586 controls	Meta-analysis and replication of a GWAS signal at *ZNF804A*	Meta-analysis and replication of a GWAS signal at ZNF804A
Wong, E.H. et al. 2013^43^	SCZ	GWAS: 498 SCZ cases and 2025 controls; Replication: 1027 cases and 1,005 controls	GWAS	Xq28 (*RENBP*, *MECP2*, and *ARHGAP4*) as susceptible loci of SCZ
Wang, Q. et al. 2013^66^	Gray matter (GM) volume in SCZ	74 first-episode treatment-naïve patients with SCZ and 51 controls	GWAS with GM volume as phenotype of SCZ	3 genes (*TBXAS1*, *PIK3C2G* and *HS3ST5*) may predict changes of GM volume in SCZ.
He, K. et al. 2013^52^	SCZ and MDD	1235 cases with SCZ,1045 with MDD and 1235 healthy controls	Replication of European GWAS signal at *CACNA1C*	*CACNA1C* as a risk gene for SCZ and MDD
Zheng, F. et al. 2014^45^	SCZ	5897 schizophrenic patients and 6323 controls; 8222 SCZ cases and 24,661 healthy controls for meta-analysis	Replication and Meta-analysis of European GWAS signal at *CACNA1C*	*CACNA1C* associated with SCZ
Luo, X. et al. 2014^54^	SCZ	In multiple independent populations (a total of 130,623 subjects)	Replication for two large-scale GWA expression studies	*CAMKK2* may have roles in SCZ susceptibility
Yuan, J. et al. 2015^47^	SCZ	506 SCZ cases and 522 healthy controls,	Meta-analysis of European GWAS signal at *MIR137*	Negative
Luo, X. et al. 2015^63^	SCZ	Combined discovery and replication sample comprising 44,123 individuals	Meta-analysis of single-marker at *ZNF323*	*ZNF323* is a susceptible gene of SCZ
Li, Z. et al. 2015^49^	SCZ	3585 patients with SCZ and 5496 controls of Han Chinese	Replication of a GWAS signal at *MIR137* -mediated pathway	Two target genes of *MIR137* (*ITIH3/4* and *CALN1*) associated with SCZ
Su, L. et al. 2016^44^	SCZ	700 SCZ patients and 700 controls (Zhuang: 300, Han: 400)	Replication of European GWAS signal at *MAD1L*	*MAD1L*1 associated with SCZ in Chinese Han
Xiao, X. & Li, M. 2016^42^	SCZ	3977 cases and 5589 controls of East Asian origin	Replication of previous GWAS signals	*MPC2* (*BRP44*) is associated with SCZ
Guan, L. et al. 2016^53^	SCZ	1471 SCZ cases and 1528 controls of Chinese Han	Replication of a GWAS signals at 17q25	17q25 (*TBCD* and *ZNF750*) associated with SCZ
Cohen, O.S. et al. 2016^58^	SCZ	16 affected families and five independent replication samples totaling 4017 affected and 4704 unaffected individuals in Chinese Han	Replication of a GWAS signals at *DRD2*	*DRD2* disrupts rs1076560(T) as one possible risk factor for SCZ
Yu, H. et al. 2016^69^	Antipsychotic induced weight gain (WG)	GWAS: 534 SCZ patients; Replication: 547 SCZ patients.	GWAS pharmacogenetic of side effects of antipsychotics	*PTPRD* and *GFPT2* were associated with WG of antipsychotic treatment
Liu, J. et al. 2016^55^	Influence of positive selection on GWAS- identified SCZ risk SNPs	Two datasets: one is a summary list of the published GWAS data for SCZ, the other is PGC2 dataset.	GWAS-identified SCZ risk SNPs at *TSPAN18* variants for samples of European and East Asian descent	*TSPAN18* rs11038172 protective allele has experienced recent Darwinian positive selection in East Asians
Liu, B. et al. 2016^67^	Cortical gyrification	Discovery dataset (*N* = 315); Replication dataset (*N* = 357)	Polygenic risk on cortical gyrification	Genetic risk for schizophrenia to cortical morphology
Yu, H. et al. 2016^56^	SCZ and meta-analysis	GWAS: 4384 SCZ cases and 5770 controls; Replication: 4339 SCZ cases and 7043 controls.	GWAS and meta- analysis in Chinese Han; Polygenic risk score (PRS) using PGC data	2p16.1 (*VRK2*), 6p22.1 (*GABBR1*) and 10q24.32 (*AS3MT*, *ARL3*) associated with SCZ in Chinese Han; PRS (R2: 1.7-5.7%).
Liu, C. et al. (in the press)^57^	GWAS pathway-based analysis in SCZ across 3 populations	5033 SCZ cases and 5332 controls. [Discovery: 972 cases and 1248 controls or European American (EA); 1125 cases and 1034 controls of Chinese Han (CH); 896 cases and 906 controls of African American (AA); Replication: 879 cases and 1132 controls of EA; 454 CH cases and 411 controls of CH;]	255 KEGG pathways based on GWAS data across three populations.	Five pathways (serotonergic synapse, ubiquitin mediated proteolysis, hedgehog signaling, adipocytokine signaling and renin secretion) were shared across all three populations.

^a^ We just enrolled the studies with more than 500 pairs of cases and controls in Han Chinese population

Both studies also supported previous findings from European populations revealing that the region of chromosome 6, associated with MHC,^7–9^ may be involved in schizophrenia in Chinese populations as well.^35, 36^ The replication of the *MHC* locus and genes in this region is of great potential significance, suggesting vital etiological immune system mechanisms for this disease that need further consideration (Cyranoski D, Nature News 2011; http://www.nature.com/news).

However, in 2013, Ma et al. did not replicate the findings of the two above mentioned GWASs in a sample of 976 unrelated schizophrenia cases and 1043 control subjects from Central China regions.^37^ Other studies have also reported different results than the above mentioned GWASs in Han Chinese or Europeans.^38–53^ The differences may reflect a relatively small power or sample size or high heterogeneity across subpopulations. Several other studies have attempted to replicate the findings of the two above mentioned GWASs in Han Chinese; however, the results remained inconsistent.

Some other recent studies have revealed surprising differences in GWAS-identified susceptibility genes or loci between European and East Asian populations. Luo et al. (2014) examined 30 GWAS-identified significant risk SNPs in Europeans and 10 SNPs in Han Chinese individuals; however, none of these genes was shared between these two geography regional populations.^54^ Liu et al. explored the evolutionary history of the tetraspanin 18 (*TSPAN18*) gene and subsequently observed the potential effects of a recent Darwinian-positive selection on the protective allele of rs11038172 in East Asians.^55^ These researchers deduced that natural selection may help to explain the strong genetic heterogeneity in schizophrenia risk and previous inconsistent association results for schizophrenia in both Europeans and East Asians.

Guan et al. assessed the initial GWAS and replicated 26 genetic variants in an independent sample of 1471 cases with schizophrenia and 1528 controls, identifying common variants on 17q25 and gene-gene interactions conferring risk of schizophrenia in Han Chinese. These authors also used the expression dataset to link tubulin-folding cofactor D (*TBCD*) and zinc finger protein 750 (*ZNF750*) mutations to disease susceptibility and the transcript levels in human brain tissues.^53^

### Comparison of GWAS-identified common polymorphisms of schizophrenia between Han Chinese and European populations

Notably, there may be other factors reflecting the differences of GWAS-identified significant genes or loci for schizophrenia among subpopulations, such as clinical heterogeneity, relative small sample size, different genotyping methods, etc. In fact, Yu et al. recently completed a meta-analysis based on GWAS data from China in a relatively large sample (GWAS: 4384 cases and 5770 controls; Replication: 4339 cases and 7043 controls).^56^ These authors further used the PGC2 GWAS data to evaluate the polygenic risk scores (PRS) to compare the overall patterns of the results from the PGC schizophrenia analysis (discovery sample) with the results from independent Chinese analyzes (target sample). The Genome-wide Complex Trait Analysis (GCTA) was also used to estimate the percentage of phenotypic variance explained by common SNPs in a Chinese population. Both PGC and Yu’s studies identified several chromosome loci, including 2p16.1 (vaccinia related kinase 2, *VRK2*), 6p22.1 (gamma-aminobutyric acid type B receptor subunit 1, *GABBR1*) and 10q24.32 (arsenite methyltransferase, *AS3MT;* ADP ribosylation factor-like GTPase 3, *ARL3*), that might be susceptibility loci for schizophrenia in both European and Han Chinese subpopulations.^56^ Functional exploration suggested that VRK2 and ARL3 may play important roles in neurodevelopment.^56^ Without reference GWAS dataset in Han Chinese, Yu et al. used the PGC2 dataset to calculate a polygenic risk score (PRS) and found the PGC PRS valid to sample from Chinese (*R* ^*2*^ = 1.7–5.7%).^56^ The use of meta-analyses or mega-analyses, including much larger sample sizes with high clinical quality (at least 50,000 ~ 100,000 pairs of schizophrenia patients and controls) across European and Asian populations or subpopulations, may explain the difference between populations. Table 3 showed the comparison of single-marker association results of schizophrenia in Chinese population and PGC2 sample. Researchers have identified the sharing of susceptible genes, such as *MHC*, *MIR137*, *ZNF804A*, *VRK2*, and *AS3MT*, across European and East Asian populations.

**Table 3:** Comparison of single-marker association results of schizophrenia in Chinese population and PGC2 sample

Chr	Position	SNP	A1	A2	Gene	Chinese population			PGC2 GWAS	
						Authors and years	OR	*P*	OR	*P*
6	27248931	rs6932590	C	T	POM121L2	Li, et al. 2010^48^	0.76	9.60E-04	1.12942	1.02E-20
6	32172993	rs3131296	A	G	NOTCH4		0.68	1.29E-06	NA	NA
6	30321732	rs3130375	A	C	RPP21		0.69	1.76E-05	NA	NA
18	53149021	rs2958182	A	T	TCF4		0.78	3.64E-06	0.95801	1.43E-04
13	42775097	rs1170099	A	G	DGKH	Zeng, et al. 2011^38^	1.61	5.00E-04	1.01298	6.55E-01
6	28215446	rs1233710	T	C	ZKSCAN4	Yue, et al. 2011^15^	0.79	4.76E−11	1.03759	1.05E-01
6	28227604	rs1635	T	G	NKAPL		0.78	6.91E−12	1.04519	4.38E-02
6	28250913	rs2142731	A	G	PGBD1		0.79	5.14E−10	NA	NA
11	44843134	rs11038167	A	C	TSPAN18		1.29	1.09E−11	0.97229	4.12E-01
11	44855597	rs11038172	A	G	TSPAN18		1.25	7.21E−10	1.0014	9.68E-01
11	44863818	rs835784	A	G	TSPAN18		1.27	2.73E−11	1.01298	2.23E-01
1	167903079	rs10489202	A	C	BRP44	Shi, et al. 2011^16^	1.23	9.50E−9	1.00995	4.27E-01
8	38031345	rs16887244	G	A	LSM1		0.84	1.27E−10	1.05527	1.51E-05
8	38133793	rs1488935	T	C	WHSC1L1		0.85	5.06E−9	0.94129	2.17E-06
2	185778428	rs1344706	T	G	ZNF804A	Li, et al. 2012^39^	1.03	2.60E-01	1.07165	1.27E-10
10	62085337	rs10761482	C	T	ANK3	Yuan, et al. 2012^50^	1.45	7.00E-03	0.96117	1.25E-03
10	62179812	rs10994336	C	T	ANK3		1.40	1.00E-04	1.03015	1.42E-01
1	167903079	rs10489202	G	T	BRP44	Ma, et al. 2013^37^	1.10	2.60E-01	1.00995	4.27E-01
6	28215446	rs1233710	C	T	ZKSCAN4		0.94	6.20E-01	1.03759	1.05E-01
6	28250913	rs2142731	T	C	PGBD1		1.04	8.33E-01	NA	NA
6	28227604	rs1635	G	T	NKAPL		0.97	4.62E-01	1.04519	4.38E-02
8	38133793	rs1488935	G	A	WHSC1L1		1.01	5.74E-01	0.94129	2.17E-06
8	38031345	rs16887244	A	G	LSM1		1.02	4.77E-01	1.05527	1.51E-05
11	44855597	rs11038172	G	A	TSPAN18		0.94	1.83E-01	1.0014	9.68E-01
11	44843134	rs11038167	C	A	TSPAN18		0.94	9.30E-02	0.97229	4.12E-01
11	44863818	rs835784	G	A	TSPAN18		0.88	1.38E-01	1.01298	2.23E-01
6	28215641	rs2235359	C	A	ZKSCAN4	Zhang, et al. 2013^46^	1.22	6.77E-01	1.03221	3.83E-02
6	28222980	rs2185955	G	A	ZKSCAN4		1.23	6.22E-01	1.03179	4.04E-02
6	28223731	rs12214383	G	A	NKAPL	Zhang, et al. 2013^46^	1.17	1.72E-01	0.93641	9.93E-10
6	28227436	rs12000	G	A	NKAPL		0.81	1.90E-03	1.00803	5.44E-01
6	28250236	rs1150724	G	A	PGBD1		1.19	1.06E-01	0.927	1.72E-11
6	28253532	rs1150722	G	A	PGBD1		0.61	3.00E-04	NA	NA
6	28264681	rs3800324	A	G	PGBD1		0.80	9.98E-02	1.01664	4.68E-01
6	28269663	rs1997660	A	G	PGBD1		1.19	1.11E-01	1.08296	4.84E-12
11	44843134	rs11038167	A	C	TSPAN18	Yuan, et al. 2013^51^	1.03	6.77E-01	0.97229	4.12E-01
11	44855597	rs11038172	A	G	TSPAN18		1.07	2.59E-01	1.0014	9.68E-01
11	44863818	rs835784	A	G	TSPAN18		1.21	4.97E-03	1.01298	2.23E-01
2	185778428	rs1344706	T	G	ZNF804A	Li, et al. 2013^40^	1.06	1.00E-01	1.07165	1.27E-10
2	185802243	rs1366842	C	T	ZNF804A	Li, et al. 2013^41^	1.13	4.00E-03	0.94904	1.24E-06
X	153207545	rs2269372	A		RENBP	Wong, et al. 2013^43^	1.31	3.98E-08	1.03334	3.71E-03
12	2345295	rs1006737	A	G	CACNA1C	He, et al. 2013^52^	1.38	1.40E-03	1.09856	1.09E-16
12	2344960	rs2007044	G	A	CACNA1C	Zheng, et al. 2014^45^	1.08	5.30E-03	0.91247	2.63E-17
12	2345295	rs1006737	A	G	CACNA1C		1.16	1.08E-02	1.09856	1.09E-16
12	2350401	rs882195	G	C	CACNA1C		1.04	1.62E-01	1.0835	8.33E-14
12	2402246	rs1024582	T	C	CACNA1C		1.18	6.20E-03	1.10087	3.40E-17
1	98502934	rs1625579	T	G	MIR137	Yuan, et al. 2015^51^	1.09	5.12E-01	1.12086	8.45E-17
3	52855229	rs2239547	G		ITIH3/4	Li, et al. 2015^49^	0.81	1.17E-10	1.05887	1.44E-06
7	71786721	rs2944829	A		CALN1		0.85	9.97E-09	0.9559	2.65E-05
7	122266911	rs2192017	A		CADPS2		1.10	1.61E-04	0.98708	2.21E-01
10	105325170	rs10748844	T		NEURL		1.09	2.96E-04	1.00381	7.19E-01
12	2499626	rs2887780	C		CACNA1C		0.90	2.25E-05	0.97336	2.07E-02
7	2004421	rs12666575	T	C	MAD1L1	Su, et al. 2016^44^	0.87	6.60E-02	0.92951	2.48E-11
1	11856378	rs1801133	T	C	MTHFR	Guan, et al. 2016^53^	0.977	7.97E-01	1.00682	5.51E-01
1	167903079	rs10489202	G	T	BRP44		1.081	3.48E-01	1.00995	4.27E-01
1	167973976	rs1060041	C	T	DCAF6		1.112	1.98E-01	1.01106	3.78E-01
1	168091031	rs11586522	C	A	GPR161		1.067	4.11E-01	1.00813	5.16E-01
3	152473776	rs1381094	A	G	P2RY1		0.860	1.45E-01	0.97424	5.49E-02
4	14171487	rs3111810	C	T	RP11-669M16.1		1.005	9.99E-01	1.0016	8.88E-01
6	26158079	rs7749823	A	C	HIST1H2BD		0.982	9.75E-01	1.18329	7.30E-23
6	28215446	rs1233710	C	T	ZKSCAN4	Guan et al. 2016^53^	0.930	3.26E-01	1.03759	1.05E-01
6	28227604	rs1635	G	T	NKAPL		0.933	3.49E-01	1.04519	4.38E-02
6	28250913	rs2142731	T	C	PGBD1		0.967	6.89E-01	NA	NA
6	28454221	rs6927023	G	A	LOC101928950		1.123	3.52E-01	NA	NA
6	29023214	rs2394514	T	C	LOC100129636		1.090	3.17E-01	NA	NA
6	29704400	rs1736913	G	A	HLA-F		0.924	2.81E-01	NA	NA
7	95068684	rs6978425	G	T	PON2		1.126	1.35E-01	1.01056	3.49E-01
8	38031345	rs16887244	A	G	LSM1		0.985	8.61E-01	1.05527	1.51E-05
8	38133793	rs1488935	G	A	WHSC1L1		1.01	9.16E-01	0.94129	2.17E-06
9	107598740	rs2065412	T	C	ABCA1		0.937	5.79E-01	1.00351	7.46E-01
11	44843134	rs11038167	C	A	TSPAN18		1.008	9.50E-01	0.97229	4.12E-01
11	44855597	rs11038172	G	A	TSPAN18		0.917	2.83E-01	1.0014	9.68E-01
11	44863818	rs835784	G	A	TSPAN18		0.991	9.22E-01	1.01298	2.23E-01
12	112110489	rs3782886	A	G	BRAP		0.991	9.40E-01	NA	NA
17	72042737	rs11077721	A	G	RP11-101O21.1		0.886	9.90E-02	NA	NA
17	80790442	rs3744165	C	A	TBCD, ZNF750		1.211	5.80E-02	0.99362	6.52E-01
17	80897492	rs8073471	A	G	TBCD		1.351	4.00E-03	0.99611	8.02E-01
18	47503579	rs4939924	T	C	MYO5B		0.944	6.52E-01	1.0141	2.10E-01
20	10030931	rs656111	C	A	ANKEF1		1.010	9.14E-01	1.02532	2.09E-02
11	113283688	rs1076560	T		DRD2	Cohen, et al. 2016^58^	1.10	4.00E-03	0.99591	7.72E-01
1	11839923	rs12561919	T	C	C1orf167	Yu, et al. 2016^56^	0.702	7.01E-08	0.980	1.81E-01
2	58316814	rs1051061	G	A	VRK2		0.826	7.11E-10	0.949	1.26E-06
3	119750146	rs10433339	A	G	GSK3B		0.827	7.18E-09	1.000	9.78E-01
5	165362010	rs58189929	T	C	TENM2		1.180	5.33E-08	0.997	8.05E-01
5	165424808	rs10077591	T	G	TENM2		1.193	5.53E-08	1.000	9.76E-01
6	29530974	rs115070292	G	A	GABBR1		0.710	1.31E-09	NA	NA
8	3219559	rs13268772	T	G	CSMD1		1.191	1.52E-08	1.004	7.07E-01
10	104654577	rs59083960	C	T	AS3MT		1.223	9.91E-07	NA	NA
10	104456686	rs10883765	C	T	ARL3		1.185	5.88E-08	1.098	1.61E-07
11	45323853	rs10883795	T	C	SYT13		1.184	4.11E-08	1.084	2.02E-12
11	45354539	rs4073405	C	T	SYT13		1.252	9.04E-10	1.030	9.35E-03
17	55825701	rs7112723	G	A	CCDC182	Yu, et al. 2016^56^	1.180	2.28E-07	1.034	1.40E-02
22	45570969	rs12949688	T	C	NUP50		0.858	7.88E-07	0.980	5.50E-02

All positions are relative to UCSC hg19

*Chr.* chromosome, *OR* odds ratio, *A12* reference and alternate allele, *NA* not available

In further analyzes, Liu et al. used the GWAS dataset from three distinct populations (European Americans, Han Chinese, and African Americans) to explore potentially shared pathways.^57^ These authors found that five pathways (serotonergic synapse, ubiquitin-mediated proteolysis, hedgehog signaling, adipocytokine signaling and renin secretion) were shared in the risk of schizophrenia across all three populations.^54^

Moreover, Cohen et al. primarily focused on the association of typical dopamine receptor D2 (*DRD2*) with schizophrenia.^58^ These studies enrolled 16 affected families and five independent replication samples, totaling 4017 affected and 4704 unaffected individuals in Han Chinese populations. They deduced that *DRD2* disrupts rs1076560 (T allele) as a potential risk factor for schizophrenia in multiple subpopulations of Han Chinese.

### Rare CNVs of schizophrenia in Han Chinese

Except for common polymorphisms, increasing studies have also demonstrated that rare variations also contribute to schizophrenia susceptibility and the CNVs explain a proportion of missing heritability. However, only few studies of CNVs in Han Chinese population have been reported to date. For example, Zhao et al. screened deletions at 15q11.2 in 2058 schizophrenia patients and 3275 normal controls in Han Chinese population and then validated deletions. They identified that rare CNVs at 15q11.2 are associated with schizophrenia, and found a significant increase of deletions in cases over controls.^59^ Li et al. performed a large-scale genome-wide CNV analysis on 6588 patients with schizophrenia and 11,904 control subjects of Han Chinese population. They validated several CNVs identified in European population, including duplications at 16p11.2, 15q11.2-13.1, 7q11.23, and VIPR2 and deletions at 22q11.2, 1q21.1-q21.2, and NRXN1. Furthermore, they identified three new potential loci: duplications at 1p36.32, 10p12.1, and 13q13.3. These findings provide further support for the role of CNVs in the etiology of schizophrenia in Han Chinese population.^34^

### Progress on other genetic markers of schizophrenia in Han Chinese

miRNA or methylation studies have also been reported in the Han Chinese population. For example, Wei et al. reported that the up-regulation of miR-130b and miR-193a-3p may be state-independent biomarkers for schizophrenia.^60^ Zhang et al. showed that the dysregulation of miRNA systems undermines the inhibitory effects of miRNAs, resulting in the abnormal up-regulation of genome transcription in the development of schizophrenia.^61^ This same group also reported that transcriptional factors and microRNAs (miRNAs) may contribute to the development of schizophrenia and might also be relevant to the clinical treatment of the disease.^62^ Based on GWAS datasets, Luo et al. verified the association of calcium/calmodulin (CAM)-dependent protein kinase kinase 2 (*CAMKK2*) with schizophrenia and subsequently observed that the T allele of rs1063843 is associated with a lower expression level of CAMKK2 in this disease.^54^ Luo et al. further explored the GWAS and expression data and observed that zinc finger protein 323 (*ZNF323*) may have brain expression Quantitative Trait Loci effects as a novel risk gene of schizophrenia. Moreover, ZNF323 showed positive selection based on compensatory advantage on pulmonary function.^63^ Based on GWAS datasets, researchers deduced that genetic markers of human evolution may be enriched in schizophrenia, which may well explain the differences of GWASs among ethnicities or subpopulations.^64^

However, studies on clinical phenotypes or endo-phenotypes could offer additional clues or suggestions for the mechanisms of complex diseases. Ma et al. suggested that the GWAS-identified risk gene, methionine sulfoxide reductase A (*MSRA*), may be associated with fluid intelligence in schizophrenia.^65^ Fluid intelligence is the capacity to reason and solve novel problems, independent of any knowledge from the past which usually be assessed with Cattell’s Culture-Free Intelligence Test (CCFIT). Wang et al. reported that reduced gray matter (GM) volume was associated with polymorphisms in thromboxane A synthase 1 (*TBXAS1*), phosphatidylinositol-4-phosphate 3-kinase catalytic subunit type 2 gamma (*PIK3C2G*) and heparan sulfate-glucosamine 3-sulfotransferase 5 (*HS3ST5*) in first-episode treatment-naïve patients with schizophrenia.^66^ Liu et al. evaluated the effects of polygenic risk on cortical gyrification and provided some implications regarding differences in the genetic risk of individuals for schizophrenia to cortical morphology and brain development in Han Chinese population.^67^ Furthermore, some GWASs have been conducted to assess treatment or side effects in Han Chinese populations.

Some GWASs with limited sample sizes have led to a potential trend in precision medicine for antipsychotic therapy^68, 69^. Liou et al. suggested that rs28362691 in nuclear factor kappa B subunit 1 (*NFKB1*) might be involved in the development of treatment refractory schizophrenia in Han Chinese individuals.^68^ Yu et al. reported that protein tyrosine phosphatase, receptor type D (*PTPRD*) and glutamine-fructose-6-phosphate transaminase 2 (*GFPT2*) polymorphisms were associated with the weight gain effects of atypical antipsychotic medications.^69^ Both *PTPRD* and *GFPT2* have been reported as susceptible genes of diabetes, suggesting a novel mechanism for weight gain induced through antipsychotic therapy. Thus, additional large-scale pharmacogenomics studies should be completed.

## Disscussion

### Sample sizes for GWASs and for pharmacogenomics

For international GWASs, the sample size of schizophrenia as the primary phenotype has been enlarged from 320 cases in the first GWAS to 35,476 cases with schizophrenia in the PGC2^4, 11^, and will be up to 100,000 cases in the PGC3 in the future^22^.

In a genome-wide pharmacogenomics study, McClay et al. reported 738 subjects with DSM-IV schizophrenia who took part in the Clinical Antipsychotic Trials of Intervention Effectiveness.^70^ To date, few genome-wide pharmacogenomics study in Han Chinese population has been published. Most of the pharmacogenetic studies focus on some candidate genes or pathways. Yu et al. used 534 patients in discovery sample to explore the body antipsychotic-induced weight gain.^69^ Yue et al. has just completed a pharmacogenomics study including 3003 patients with schizophrenia treated with 7 antipsychotic drugs for 6 weeks in Han Chinese population (unpublished data).

### Genetic architecture of the schizophrenia disease in Chinese populations

Population structure can potentially cause inaccurate associations. Therefore, it is critical to understand the genetic structure of a population. The Han Chinese population is the largest ethnic group in the world, composing 20% of the world population, the Han Chinese population constitute more than 90% of China’s population. However, the Han Chinese has been largely underrepresented in most of the GWASs. Considering modern human origins, the “Out-of-Africa” hypothesis has been supported by genetic and archeological evidences, however, the scenario of colonization of East Asia need further clarification. Genetic evidence has provided some indication that East Asia humans may migrate or come from through the following two roads: along the coast to south and Southeast Asia (the southern road), or through the Middle East to Central Asia (the northern road). As for Han Chinese substructure, Xu et al. reported that the Han Chinese population was intricately sub-structured, with the main observed clusters corresponding roughly to northern Han, central Han, and southern Han.^71^ Moreover, using over 350,000 SNPs in over 6000 Han Chinese samples from ten provinces of China, Chen et al. explored Chinese population structure across the geographical locations, and examined the potential magnitude of Chinese population stratification. They finally revealed a one-dimensional “north-south” population structure and a close correlation between geography and the gene structure of the Han Chinese.^72^ Actually, in the 1000 Genomes project, the Chinese population was categorized under the generic terms of “Southern Han” and “Northern Han”. Therefore, in the genetic association studies Han of Chinese population, it should be ensured that homogeneous samples were recruited or analyzed. For example, to reduce the effects of population structure, Yu et al. performed a stratified GWAS of schizophrenia in Han Chinese population based on the North-South sub-population structures, and then combined the GWAS results by meta-analysis.^69^ Population structure analysis should be done before the analysis using Multidimensional Scaling Analysis or Principal Component Analysis, and should be used as a covariate. Moreover, the inflation factor or linkage disequilibrium-based regression score could also be performed to examine the effects of population structure on association results.

### Heritability studies of schizophrenia in Han Chinese

In the 1970s, the Shanghai Polygenic Inheritance Collaborative Research Group investigated the heritability of schizophrenia in Chinese individuals using classical genetic methods (i.e., family surveys, twin and adoption studies) and estimated a heritability of 82.9% in the Chinese population.^73^ Subsequent epidemiologic study reported heritability for schizophrenia of 63 to 78% in Chinese individuals and heritability of 70% in Chinese children (Lou, 1983).^74^ Additionally, we previously estimated the heritability of schizophrenia due to common GWAS SNPs. Assuming a population risk of 0.01, we estimated from GCTA software that 40.2% (s.e. of 0.02) of the total variation was explained by common SNPs across the genome.^56^ It suggested that the variance in liability estimated from GCTA accounts for over 51.3% of the observed heritability (0.40/0.78). However, this SNP-based heritability analysis is still inaccurate, and a future twin meta-analysis could better estimate the heritability of schizophrenia in the Chinese population. Nevertheless, this analysis revealed that common SNPs also make substantial contributions to the risk for schizophrenia in the Han Chinese population, and there are additional schizophrenia susceptibility loci yet to be discovered, including both common and rare variants.

### Support for GWASs of schizophrenia in Han Chinese in the future

In recent decades, the central government of China has launched several large projects for mental disorders. More than 140 new studies involving psychiatric genetic studies were funded through the National Natural Science Foundation of China or the Ministry of Science and Technology. The Precision Medicine Program and the National Key Research and Development Program of China on Chronic Diseases have approved four and six programs for psychiatric disorders, respectively. The Human Brain Project of China will also be launched in 2017.

The “National 686 Project” for the management of the severe psychiatric disorders, launched by the central government of China, has enrolled 5.4 million patients with severe psychiatric disorders, including schizophrenia, mental retardation, and epilepsy with psychiatric syndromes. The first guideline on improving mental health services was released on 19 Jan 2017, according to 22 ministries and, ministerial-level departments led by the National Health and Family Planning Commission (China Daily). All of the above findings suggest that multi-omics strategies will be helpful in the future for the exploration of the genetic background or mechanism for schizophrenia in China.

To coordinate research efforts in psychiatric genetics in China, a group of Chinese and foreign investigators have established an annual “Summit on Chinese Psychiatric Genetics” to present their latest research and to discuss the current state and future directions of this field. The Summit on Chinese Psychiatric Genetics has been held four times across China, and a session conference on the XXIV World Congress of Psychiatric Genetics was conducted in Israel in Oct 2016 (www.wcpg2016.org). The famous non-governmental organizations, such as Beijing Genomics Institute (http://www.genomics.cn/), CapitalBio Corporation (http://www.capitalbio.com/) and other biotech companies have made collaborative research efforts for psychiatric disorders with multiple research institutes in China.

Furthermore, Chinese researchers also have been establishing collaborations with international fellowships or scholars working on meta-analyses or mega-analyses in much larger samples across European and Asian populations. Thus, the GWAS findings will further contribute to the exploration of the mechanisms of schizophrenia and potential treatment targets of this complex disease.

## Conclusions

Accompanied by the rapid development of international GWASs of schizophrenia, an expanded list of common risk loci were identified in Han Chinese populations. However, when the sample size or genetic statistic power increased, much more striking shared susceptible genes or loci were identified among European and East Asian populations. Moreover, there were susceptibility loci unique to Han Chinese. In conclusion, although many confounding factors may influence the results of GWASs of schizophrenia between European and Han Chinese populations or sub-populations of Han, several common or rare variations have also been identified that overlap in multi-ethnicities. Researchers have identified the sharing of susceptible genes, such as *MHC*, *MIR137*, *ZNF804A*, *VRK2*, and *AS3MT*, across European and East Asian populations. Several CNVs identified in European population have also been validated in Han Chinese, including duplications at 16p11.2, 15q11.2-13.1, 7q11.23, and VIPR2 and deletions at 22q11.2, 1q21.1-q21.2, and NRXN1.

In the future, it should be helpful to use the meta-analyses or mega-analyses that include much larger sample sizes (at least 50,000 ~ 100,000 cases of schizophrenia and a considerable number of controls) across European and Asian populations or subpopulations. Moreover, other directions may include multi-omics methods, deep sequencing of the genome, precision medicine, and further functional exploration of risk genes to explain the mechanism of the disease.
